# A Longitudinal Analytical Study on Umbilical Cord Coiling Index as a Predictor of Pregnancy Outcome

**DOI:** 10.7759/cureus.35680

**Published:** 2023-03-02

**Authors:** Gopinath Subashini, Christina Anitha, Ganesan Gopinath, K Ramyathangam

**Affiliations:** 1 Obstetrics and Gynaecology, Annaii Medical College and Hospital, Sriperumbudur, IND; 2 Radiology, Panimalar Medical College Hospital and Research Institute, Chennai, IND; 3 Obstetrics and Gynaecology, Employees State Insurance (ESI) Medical College and Post Graduate Institute of Medical Sciences and Research (PGIMSR), Chennai, IND

**Keywords:** emergency lscs, adverse outcomes, postnatal, antenatal, umbilical cord coiling index

## Abstract

Background

The umbilical cord coiling index (UCI) is usually measured sonographically during antenatal follow-up and can be used to determine the fetuses at risk of adverse outcomes.

Methodology

UCI measured antenatally and postnatally whose correlation is studied along with the association of abnormal UCI with the adverse outcomes in terms of gestational age, intrauterine growth restriction (IUGR), intra-uterine death, birth weight, sex, neonatal intensive care unit (NICU) admission, the color of the liquor, Amniotic Fluid Index (AFI), Appearance, Pulse, Grimace, Activity, and Respiration (APGAR) score at one min and five mins and mode of delivery. All parameters are tested for significant differences among UCI and a p-value < 0.05 is considered significant. The correlation of UCI measured antenatally and postnatally is tested using the spearman correlation coefficient.

Results

A strong correlation is found between antenatal UCI and postnatal UCI with r_s_ 0.9. The majority of the population had normo coiling. Hyper and hypo coiling are associated risks of emergency lower segment cesarean section (LSCS). Low birth weight is seen in 88.89% of hypo coiled patients with a p-value < 0.01. The coiling index among sex is found to be insignificant with a p-value of 0.81. Meconium-Stained Liquor (MSL) is seen in 78.5% of hyper coiled patients. IUGR is found to be associated with hypo coiling as seen in 59.2% of patients with significant p-value (< 0.01). Age, gestational age, and birth weight are found to be statistically significant between various coiling indexes with p-value < 0.05.

Conclusion

Antenatal UCI correlates with postnatal UCI and any abnormal index found can be used as a predictor of adverse perinatal outcomes and help obstetricians to monitor continuously and put the patients at risk on prophylactic measures.

## Introduction

The umbilical cord is a fascinating and complex structure that connects the placenta and fetus [1]. The umbilical cord is referred to as the 50-60 cm long coiled structure with the major blood vessels and Wharton’s jelly that has 10-11 coils from placental insertion to the fetus and is very important for fetal growth and well-being [2]. Rich sources of hematopoietic stem cells are present in the umbilical cord [3]. The umbilical cord contains one umbilical vein, two umbilical arteries, porous Wharton’s jelly, and an outer layer of amnion [4]. At term pregnancy, the blood flow to the fetus is protected by the umbilical cord. Fluid pressure within the umbilical cord is regulated by the outer amnion layer. The compression of the vessels is prevented by porous fluid-filled Wharton’s jelly [4].

To correlate with possible antenatal and perinatal complications, umbilical cord characteristics such as the number of vessels, amount of Wharton’s jelly, cord length, type of placental insertion, cord thickness, blood flow patterns, cord diameter, and coiling are analyzed [5]. The number of coils divided by the length of the umbilical cord in centimeters gives the umbilical cord index [6]. The umbilical cord coiling index (UCI) has a strong relationship with perinatal outcomes and may be used antenatally as a marker for identifying the fetus at risk [7]. Using an antenatal sonographic longitudinal view of cord vessels in several segments and dividing it by the postnatally observed number of helices, the umbilical index can be calculated [8]. To determine its correlation with pregnancy outcomes, UCI was measured and analyzed [9]. From the first trimester, using sonography the degree of cord coiling is identified [10]. The umbilical cord index mean was 0.17(0.009) coils [11]. The umbilical coiling index < 0.17 percentile is referred to as hypo coiled and the umbilical cord index > 0.37 percentile is referred to as hyper coiled. UCI between 0.17-0.37 is referred to normo coiled [12].

UCI below the 10th percentile (under coiling) and UCI above the 90th Percentile (over coiling) are associated with increased risk for adverse perinatal outcomes [13]. Umbilical under coiling is associated with fetal death, chorioamnionitis, fetal structural or chromosomal abnormalities, and a lower Appearance, Pulse, Grimace, Activity and Respiration (APGAR) score of five mins. Umbilical over coiling is associated with fetal death, Iatrogenic preterm delivery, umbilical arterial PH < 7.05, fetal structural or chromosomal abnormalities, thrombosis in fetal placental vessels, chronic fetal hypoxia/ischemia and lower weight for gestational age [14].

The current study is conducted to correlate the umbilical cord index measured antenatally using ultrasonography with that of the umbilical cord index measured postnatally and the association of this index with adverse fetal outcomes.

Objectives of the study

1. To assess the correlation between the antenatal umbilical coiling index & true umbilical coiling index at birth. 2. To study whether abnormal umbilical cord coiling index detected antenatally by ultrasonography will correlate with true umbilical coiling index at birth. 3. To determine the possibility of adverse pregnancy outcomes (gestational age at delivery, mode of delivery, the birth weight of baby, APGAR score, meconium staining of liquor & intrauterine growth restriction (IUGR). using antenatal umbilical cord coiling index. 

## Materials and methods

Study design

The study was designed as a longitudinal analytical study for 18 months. The study was done after obtaining institutional ethical committee clearance. 

Inclusion criteria consisted of a) booked singleton pregnancy irrespective of parity, b) reliable gestational age between 18 to 23 weeks at the time of sonography, c) normal amniotic fluid index, d) presence of three-vessel umbilical cord, e) planned delivery at this institution. 

The exclusion criteria consisted of a) multiple pregnancies, b) fetal congenital anomalies, and c) maternal complications that interfere with fetal growth (such as diabetes mellitus, pregnancy-induced hypertension, and anemia complicating pregnancy).

The sample size was identified to be 200. Qualitative analysis was done, n=4pq/lA2 (n-sample size, p-prevalence (positive character), q- 100-p (negative character). 1-allowable error (20%). According to the study (Nivedita et al.), the prevalence of abnormal coiling is 34%. So, p=34%, q=66, 1=6.8. n=194

Procedure

Pregnant women who came to the obstetrics and gynecology (OBG) department of our tertiary care medical college hospital, and met our inclusion & exclusion criteria were explained about the project in detail and informed consent was obtained from the patients who expressed an interest in participating. The study variables were collected from the participants and analyzed. Antenatally measured umbilical cord coiling index is referred to as UCI1 and the umbilical cord coiling index measured postnatally is referred to as UCI2. Confidentiality was ensured by not collecting their personal, social, and economic details.

Statistical analysis

Antenatal umbilical coiling index (UCII) and postnatal umbilical coiling index (UCI2), were considered as primary outcome variables. Gestational age at delivery, mode of delivery, birth weight (baby), APGAR score, and intrauterine growth restriction (IUGR) were considered secondary outcome variables. For normally distributed quantitative parameters the mean values were compared between study groups using analysis of variance (ANOVA) (more than two groups). Categorical outcomes were compared between study groups using the Chi-square test, p-value < 0.05 was considered statistically significant. Data were analyzed by using CoGuide software (BDSS Corp. Released 2020. coGuide Statistics software, Version 1.0, Bangalore, India: BDSS corp) [15].

## Results

A total of 200 subjects were included in the final analysis.

**Figure 1:**
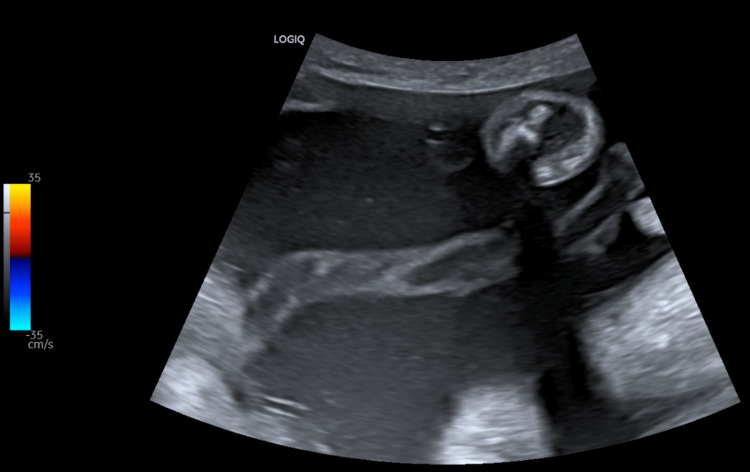
Grayscale USG image showing a free loop of umbilical cord

**Figure 2:**
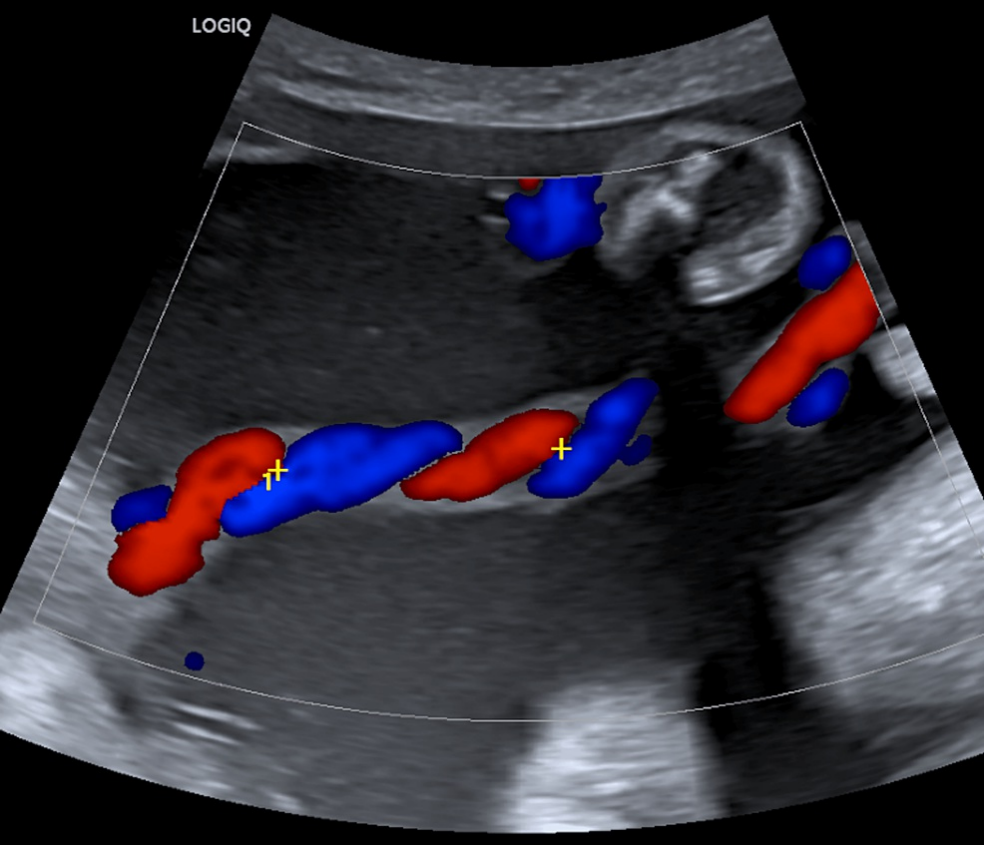
Color doppler USG image showing a free loop of umbilical cord. One coil containing 2 arteries and one vein depicted within the calipers.

In postnatal umbilical coiling index with normo coiling, hyper coiling, and hypo coiling, the majority of 140 (96.55%) participants were in normo coiling, 24 (85.71%) were in hyper coiling and 24 (88.89%) were in hypo coiling as per antenatal umbilical coiling index (Figures 1, 2) The difference in the proportion of age groups (years) between postnatal umbilical coiling index was statistically not significant with a p-value of 0.0502. The difference in the proportion of parity between postnatal umbilical coiling index was statistically not significant with a p-value of 0.3182. In postnatal umbilical coiling index with normo coiling, hyper coiling, and hyper coiling, the majority of 120 (82.76%) participants had normal vaginal delivery (NVD) mode of delivery, 20 (71.43%) had emergency lower segment caesarean section (LSCS) and 15 (55.56%) had NVD mode of delivery (Table 1).

**Table 1:** Comparison of postnatal umbilical coiling index with gestational age (weeks), intrauterine death, birth weight (kg) and Sex (Baby) in the study population (N=200) *No statistical test was applied- due to 0 subjects in the cells

Postnatal umbilical coiling index (UCI2)	Gestational age (weeks)		p-value
	Normal (Term (>=37))	Abnormal (Preterm (< 37)	
Normo coiling (N = 145)	140 (96.55%)	5 (3.45%)	< 0.001
Hyper coiling (N = 28)	24 (85.71%)	4 (14.29%)	
Hypo coiling (N = 27)	16 (59.26%)	11 (40.74%)	
Intrauterine death			
Postnatal umbilical coiling index (UCI2)	Yes	No	p-value
Normo coiling (N = 145)	0 (0.00%)	145 (100.00%)	*
Hyper coiling (N = 28)	0 (0.00%)	28 (100.00%)	
Hypo coiling (N = 27)	1 (3.70%)	26 (96.30%)	
Birth weight (kg)			
Postnatal umbilical coiling index (UCI2)	< 2.5	>=2.5	p-value
Normo coiling (N = 145)	3 (2.07%)	142 (97.93%)	< 0.001
Hyper coiling (N = 28)	9 (32.14%)	19 (67.86%)	
Hypo coiling (N = 27)	24 (88.89%)	3 (11.11%)	
Postnatal umbilical coiling index (UCI2)	Sex (Baby)		p-value
	Boy	Girl	
Normo coiling (N = 145)	77 (53.10%)	68 (46.90%)	0.8109
Hyper coiling (N = 28)	13 (46.43%)	15 (53.57%)	
Hypo coiling (N = 27)	14 (51.85%)	13 (48.15%)	

Out of 145 normo coiling in postnatal umbilical coding index, the majority of 123 (84.83%) participants were in < 37 weeks gestational age at delivery. Out of 28 hyper coiling, the majority of 24 (85.71%) participants were at 37 to 40 weeks gestational age at delivery. Out of 27 hypo coiling, the majority of 15 (55.56%) participants were in > 40 weeks gestational age at delivery (Table 2).

**Table 2:** Comparison of antenatal umbilical coiling index, age group (years), parity and mode of delivery with postnatal umbilical coiling index in the study population (N=200) *No statistical test was applied- due to 0 subjects in the cells NVD: Normal Vaginal Delivery, LSCS: Lower segment Caesarean section

Parameter	Postnatal umbilical coiling index (UCI2)			p-value
	Normo coiling (N=145)	Hyper coiling (N=28)	Hypo coiling (N=27)	
Antenatal umbilical coiling index (UCII)				
Normo coiling	140 (96.55%)	4 (14.29%)	3 (11.11%)	*
Hyper coiling	2 (1.38%)	24 (85.71%)	0 (0.00%)	
Hypo coiling	3 (2.07%)	0 (0.00%)	24 (88.89%)	
Age Group (years)				
<=25 years	65 (44.83%)	12 (42.86%)	6 (22.22%)	0.0502
26-30 years	57 (39.31%)	14 (50.00%)	12 (44.44%)	
>30 years	23 (15.86%)	2 (7.14%)	9 (33.33%)	
Parity				
Primi	66 (45.52%)	17 (60.71%)	12 (44.44%)	0.3182
Multi	79 (54.48%)	11 (39.29%)	15 (55.56%)	
Mode of delivery				
NVD	120 (82.76%)	6 (21.43%)	15 (55.56%)	*
Emergency LSCS	15 (10.34%)	20 (71.43%)	12 (44.44%)	
Vacuum	8 (5.52%)	2 (7.14%)	0 (0.00%)	
Forceps	2 (1.38%)	0 (0.00%)	0 (0.00%)	

The difference in the proportion of postnatal umbilical coiling index between Gestational age (weeks) and Birth weight (kg) was statistically significant with p-value < 0.001. Out of 27 hypo coiling, one (3.70%) participant had reported intrauterine death (Table 3).

**Table 3:** Comparison of postnatal umbilical coiling index with gestational age at delivery (weeks) in the study population (N=200) *No statistical test was applied- due to 0 subjects in the cells

Postnatal umbilical coiling index (UC12)	Gestational age at delivery (weeks)			p-value
	< 37	37-40	>40	
Normo coiling (N = 145)	5 (3.45%)	123 (84.83%)	17 (11.72%)	*
Hyper coiling (N = 28)	4 (14.29%)	24 (85.71%)	0 (0.00%)	
Hypo coiling (N = 27)	11 (40.74%)	15 (55.56%)	1 (3.70%)	

The difference in the proportion of postnatal umbilical coiling index between amniotic fluid index (AFI), IUGR, neonatal intensive care unit (NICU), and APGAR at 1 min was statistically significant with a p-value < 0.001. Out of 145 normo coiling in the postnatal umbilical coiling index, the majority of 139 (95.86%) participants had the clear color of the liquor. Out of 28 hyper coilings, the majority of 22 (78.57%) participants had MSL color of the liquor. Out of 27 hypo coiling, the majority of all of them 27 (100.00%) had the clear color of the liquor. Out of 145 normo coiling, the majority of all of them 145 (100.00%) participants were >=7 APGAR score. Out of 28 hyper coilings, the majority of 25 (89.29%) participants were >=7 APGAR score. Out of 27 hypo coiling, the majority of all of them 26 (96.30%) were >=7 APGAR score. (Tables 4, 5, 6, 7, 8).

**Table 4:** Comparison of postnatal umbilical coiling index with AFI in the study population (N=200)

Postnatal umbilical coiling index (UCI2)	AFI		p-value
	Normal	Reduced	
Normo coiling (N = 145)	141 (97.24%)	4 (2.76%)	< 0.001
Hyper coiling (N = 28)	26 (92.86%)	2 (7.14%)	
Hypo coiling (N = 27)	10 (37.04%)	17 (62.96%)	

**Table 5:** Comparison of postnatal umbilical coiling index with colour of liquor in the study population (N=200) *No statistical test was applied- due to 0 subjects in the cells

Postnatal umbilical coiling index (UCI2)	Colour of Liquor		p-value
	Clear	MSL	
Normo coiling (N = 145)	139 (95.86%)	6 (4.14%)	*
Hyper coiling (N = 28)	6 (21.43%)	22 (78.57%)	
Hypo coiling (N = 27)	27 (100.00%)	0 (0.00%)	

**Table 6:** Comparison of postnatal umbilical coiling index with IUGR in the study population (N=200)

Postnatal umbilical coiling index (UCI2)	IUGR		p-value
	Yes	No	
Normo coiling (N = 145)	1 (0.69%)	144 (99.31%)	< 0.001
Hyper coiling (N = 28)	8 (28.57%)	20 (71.43%)	
Hypo coiling (N = 27)	16 (59.26%)	11 (40.74%)	

**Table 7:** Comparison of postnatal umbilical coiling index with NICU in the study population (N=200)

Postnatal umbilical coiling index (UCI2)	NICU		p-value
	Yes	No	
Normo coiling (N = 145)	13 (8.97%)	132 (91.03%)	< 0.001
Hyper coiling (N = 28)	21 (75.00%)	7 (25.00%)	
Hypo coiling (N = 27)	16 (59.26%)	11 (40.74%)	

**Table 8:** Comparison of postnatal umbilical coiling index with APGAR score at 1 & 5 min in the study population (N=200) *No statistical test was applied- due to 0 subjects in the cells

Postnatal umbilical coiling index (UCI2)	APGAR at 1 min		p-value
	< 7	>=7	
Normo coiling (N = 145)	19 (13.10%)	126 (86.90%)	< 0.001
Hyper coiling (N = 28)	13 (46.43%)	15 (53.57%)	
Hypo coiling (N = 27)	12 (44.44%)	15 (55.56%)	
Postnatal umbilical coiling index (UCI2)	APGAR at 5 min		
	< 7	>=7	
Normo coiling (N = 145)	0 (0.00%)	145 (100.00%)	*
Hyper coiling (N = 28)	3 (10.71%)	25 (89.29%)	
Hypo coiling (N = 27)	1 (3.70%)	26 (96.30%)	

**Figure 3:**
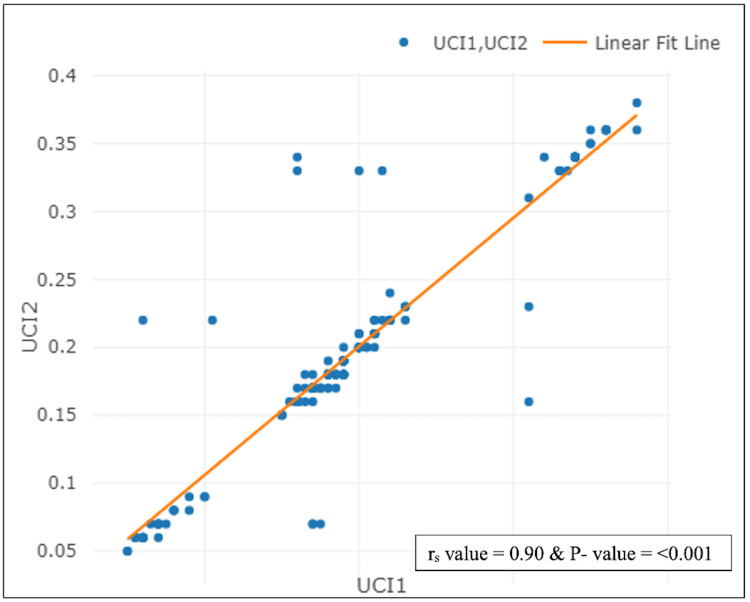
Scatter plot diagram of correlation between UCI1 with UCI2 in the study population (N=200)

The mean age (years) within normo coiling was 26.47 ± 4.09, it was 26.11 ± 3.39 in hyper coiling and it was 28.78 ± 4.11 in hypo coiling. The mean difference of age (years) in the postnatal umbilical coiling index was statistically significant with a p-value of 0.0166. The mean difference between gestational age at delivery (weeks) and birth weight (kg) in the postnatal umbilical coiling index was statistically significant with a p-value < 0.001 (Table 9). There was a strong positive correlation between the antenatal umbilical coiling index (AUCI) and with postnatal umbilical coiling index (PUCI) in the study population. (rs value: 0.90, p-value < 0.001) (Figure 3).

**Table 9:** Comparison of age (years), gestational age at delivery (weeks) and birth weight (kg) with postnatal umbilical coiling index in the study population (N=200)

Parameter	Postnatal umbilical coiling index (UCI2) (Mean ± SD)			p-value (One-way ANOVA)
	Normo coiling (N=145)	Hyper coiling (N=28)	Hypo coiling (N=27)	
Age (years)	26.47 ± 4.09	26.11 ± 3.39	28.78 ± 4.11	0.0166
Gestational age at delivery (weeks)	38.98 ± 1.07	38.31 ± 1.51	36.91 ± 2.04	< 0.001
Birth weight (kg)	2.95 ± 0.30	2.75 ± 0.51	2.20 ± 0.28	< 0.001

## Discussion

Our study reported a very strong correlation between the antenatal umbilical cord coiling index and the post-natal umbilical cord coiling index with an rs value of 0.90. However, the majority of the population had normo coiling. Hyper and hypo coiling were also seen and were associated with the risk of emergency LSCS. Low birth weight is seen in 88.89% of hypo coiled patients with a p-value < 0.01. The difference between the coiling index among sex is found to be insignificant with a p-value of 0.81. Meconium-stained liquor (MSL) is seen in 78.5% of hyper coiled patients. IUGR is found to be associated with hypo coiling as seen in 59.2% of patients with a significant p-value of < 0.01. Age, gestational age, and birth weight are found to be statistically significant between various coiling indexes with p-value < 0.05.

The majority of the population in our study are with normo coiling (72.5% as seen post-natally) which is in line with the study conducted by Chitra et al., where normo coiling was seen predominantly with 78.3% [16]. The same study reported an association of hyper coiling with MSL and hypo coiling with low birth weight which is in agreement with the current study findings [16]. Another study conducted by Aruna Biradar et al. assessed the association of UCI with adverse outcomes and reported a significant difference with a p-value < 0.01 in terms of birth weight, as seen in our study with a p-value < 0.01 [17].

A study conducted by Milani et al. reported insignificant differences among parity of the pregnancy across coiling indexes with a p-value of 0.26 which is in agreement with the current study p-value of 0.31 [18]. The study conducted by Patil et al. tested for the association of UCI with adverse outcomes that revealed a significant difference in terms IUGR with p-value < 0.01 similar to our study findings [19]. The same study also falls in line with the current study with a significant p-value of < 0.01 in terms of NICU admission. A study conducted by Kashanian et al. opposes the report of the current study in terms of low birth weight, which is seen as higher in hypo coiled group in comparison to hyper coiled in our study and vice-versa is seen in their project findings [12]. A meta-analysis conducted by Pergialiotis et al., reported findings to our study: hypocoiled UCI was associated with pre-term delivery (< 37 weeks GA), and hyper coiled UCI was associated with meconium staining [20].

Our study results are in line with other similar study results [7]. There is greater support for use of antenatal UCI in detecting at-risk fetuses in developing adverse outcomes in terms of birth weight, IUGR, MSL & APGAR score. 

## Conclusions

The longitudinal analytical study conducted to assess the correlation of the umbilical cord coiling index measured antenatally with the umbilical cord coiling index measured at birth reported a strong positive correlation. The association of abnormal umbilical cord coiling index with adverse pregnancy outcomes was found to be significant for low birth weight, oligohydramnios, meconium staining of liquor, preterm labor, IUGR, and IUD. Hence, UCI measured antenatally can be used as a predictor of adverse perinatal outcomes and help obstetricians to monitor continuously and put the patients at risk on prophylactic measures.
